# Collaborative and Structured Network for Maintenance of Mechanical Ventilators during the SARS-CoV-2 Pandemic

**DOI:** 10.3390/healthcare9060754

**Published:** 2021-06-18

**Authors:** Daniel Motta, Luiz Fernando Taboada Gomes Amaral, Bruno Caetano dos Santos Silva, Lucas de Freitas Gomes, Willams Teles Barbosa, Rodrigo Santiago Coelho, Bruna Aparecida Souza Machado

**Affiliations:** 1 Postgraduate Program MPDS/GETEC/MCTI, University Center SENAI CIMATEC, SENAI CIMATEC, Av. Orlando Gomes, 1845 Piatã, Salvador 41650-010, BA, Brazil; dmotta@fieb.org.br (D.M.); luiz.amaral@fieb.org.br (L.F.T.G.A.); bruno.silva@fieb.org.br (B.C.d.S.S.); lucas.gomes@fieb.org.br (L.d.F.G.); williams.barbosa@fbter.org.br (W.T.B.); rodrigo.coelho@fieb.org.br (R.S.C.); 2 SENAI Institute of Innovation (ISI) in Automation (CIMATEC ISI Automation), SENAI CIMATEC, Av. Orlando Gomes, 1845 Piatã, Salvador 41650-010, BA, Brazil; 3 SENAI Institute of Innovation (ISI) in Forming and Joining of Materials (CIMATEC ISI F&J), SENAI CIMATEC, Av. Orlando Gomes, 1845 Piatã, Salvador 41650-010, BA, Brazil; 4 SENAI Institute of Innovation (ISI) in Advanced Health Systems (CIMATEC ISI SAS), SENAI CIMATEC, Av. Orlando Gomes, 1845 Piatã, Salvador 41650-010, BA, Brazil

**Keywords:** mechanical pulmonary ventilators, SARS-CoV-2, maintenance, pandemic, collaborative network

## Abstract

The SARS-CoV-2 pandemic in Brazil has grown rapidly since the first case was reported on 26 February 2020. As the pandemic has spread, the low availability of medical equipment has increased, especially mechanical ventilators. The Brazilian Unified Health System (SUS) claimed to have only 40,508 mechanical ventilators, which would be insufficient to support the Brazilian population at the pandemic peak. This lack of ventilators, especially in public hospitals, required quick, assertive, and effective actions to minimize the health crisis. This work provides an overview of the rapid deployment of a network for maintaining disused mechanical ventilators in public and private healthcare units in some regions of Brazil during the SARS-CoV-2 pandemic. Data referring to the processes of maintaining equipment, acquiring parts, and conducting national and international training were collected and analyzed. In total, 4047 ventilators were received by the maintenance sites, and 2516 ventilators were successfully repaired and returned to the healthcare units, which represents a success rate of 62.17%. The results show that the maintenance initiative directly impacted the availability and reliability of the equipment, allowing access to ventilators in the public and private health system and increasing the capacity of beds during the pandemic.

## 1. Introduction

In December 2019, an outbreak of viral infection was first reported in Wuhan, China, when several patients presented symptoms of pneumonia with unknown etiology [1,2]. After conducting studies, the International Commission on Virus Classification (ICTV) classified it as severe acute respiratory syndrome coronavirus 2 (SARS-CoV-2) [3,4]. In the same period, the World Health Organization (WHO) named the disease caused by this virus COVID-19 (Corona Virus Disease 2019).

By the beginning of March 2021, a total of 120,002,856 cases of COVID-19 were confirmed worldwide, including 2,655,755 deaths, with confirmed numbers reaching 11,483,370 cases and 278,229 deaths in Brazil [5]. The high number of cases in Brazil is mainly associated with the community transmission that has rapidly established itself throughout the country since the beginning of the first case [6,7,8]. According to the Centers for Disease Control and Prevention (CDC), an increase in the number of cases will put more strain on health care resources, leading to more hospitalizations, and potentially more deaths [9].

Common symptoms of SARS-CoV-2 infection include respiratory symptoms, fever, cough, shortness of breath, and dyspnea. In cases where there is a moderate to severe clinical course, the infection can cause pneumonia, severe acute respiratory syndrome, renal failure, and even death [10,11]. Patients diagnosed with COVID-19 who show moderate to severe signs are treated symptomatically, along with oxygen therapy. In cases where patients progress to respiratory failure and become resistant to oxygen therapy, the use of mechanical ventilation is necessary and essential [12,13]. It is estimated that at least 5% of patients with COVID-19 need care in the intensive care unit (ICU), where most need mechanical ventilation [14,15]. In the pandemic context, it is noteworthy that the lack of personal protective equipment and intensive ventilators in many countries was a crucial factor in worsening the situation and creating difficulties in “flattening the curve”, as well as controlling the number of deaths [16,17,18]. The current context still brings therapeutic challenges and logistical uncertainties.

It is already well established that respiratory failure is the most prevalent and feared complication of pneumonia caused by SARS-CoV-2, usually causing hypoxemia. In this case, 10 to 20% of cases become difficult to control, necessitating the use of frequent mechanical ventilation for weeks or months [11,19]. The COVID-19 pandemic, with the large volume of cases, brought large-scale use of artificial ventilators and the consequent scarcity of these resources to meet a demand never before seen. Due to the inability of manufacturing companies to meet market demand, equipment prices rose exponentially.

The growing daily increase in the number of cases of COVID-19 and the need for intensive care have led to resource limitations in meeting the demand for mechanical ventilators for the treatment of severe acute respiratory syndrome worldwide. This has increased the search for new alternatives, including efficiently increasing production capacity [16,20]. There were growing concerns in many countries, including Italy and Brazil, that the number of mechanical ventilators would not meet the high demand, and that patients would be unassisted due to the lack of available equipment [21]. Despite efforts to expand the availability of lung ventilators around the world, it should be noted that the absence of these medical devices directly compromised the clinical development of patients, resulting in even more deaths [22]. In Italy, these circumstances raised a critical issue: the demand for ventilators and other intensive treatments far exceeded the supply, and at the time when the country was considered the epicenter of the pandemic, criteria were established [23] that guided rationing decisions for the “appropriate” use of available ventilators [21].

Several countries, particularly those with fewer resources, had many difficulties in acquiring equipment, reflected in falling assistance and increased mortality, as was the case in Brazil [24,25], where less than half of the ventilators purchased by state governments since the beginning of the pandemic were delivered by contracted companies [26,27]. The Brazilian scenario regarding the number of beds and availability of mechanical ventilation support during the pandemic has been a concern [28]. In January 2020, Brazil had 29,891 ICUs linked to the public (SUS; 14,094) and private (15,797) systems. Regarding mechanical ventilation, SUS claimed to have only 40,508 units. However, reliable data on the actual situation of the equipment are scarce due to the lack of registration in databases [29].

This context demanded fast, assertive, and effective actions to minimize the health crisis in Brazil, which quickly became the epicenter of the pandemic in Latin America [30,31]. Thus, an alternative to increasing ventilation capacity not only for COVID-19, but for all patients requiring mechanical ventilation, was to maintain existing and out-of-service ventilators in public and private healthcare units in the country [32,33]. 

**Figure 1:**
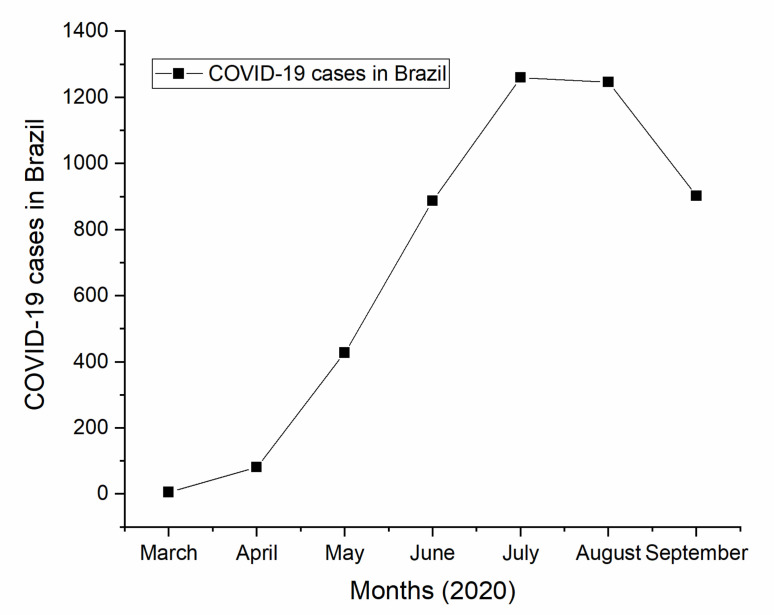
Evolution of COVID-19 cases in Brazil from March to September 2020.

In April 2020, estimates of Ministry of Health of Brazil indicated that the country would need about 20,000 additional ventilators, and at the peak of the pandemic, 2000 new ventilators would be needed per week [34]. According to the evolution of cases registered in Brazil, as can be seen in Figure 1, between the months of March and September of 2020, an increase of the bed occupancy rate of ICUs in Brazil and, consequently, in the number of ventilators needed, was observed [35,36,37].

In this work, we provide a brief overview of the rapid structuring of a collaborative network for the maintenance of mechanical ventilators during the SARS-CoV-2 pandemic. This network was named Initiative + Maintenance of Ventilators (I+M). In addition, we present the main results obtained and the social contribution of allowing access to this equipment in the public health system in Brazil, consequently increasing the bed capacity during the pandemic. This work has very important social relevance for Brazil in the context of the epidemic caused by COVID-19, which is still creating a high demand for respiratory care. Although the I+M represents a national network, this work presents specific results achieved by SENAI CIMATEC (Salvador, Bahia, Brazil). This institution was the first and the main site of the I+M and was considered a reference for the entire Brazilian network, and therefore is the so-called Reference Unit in this work. 

## 2. Materials and Methods

### 2.1. Mechanical Ventilator

A mechanical ventilator is considered an artificial external organ, originally designed to provide oxygenation and respiratory support for patients who cannot breathe enough on their own due to impaired lung system functions [38], a condition called respiratory failure [39,40]. Modern ventilators are exclusively positive pressure and include complex software to facilitate different modes of ventilation and diagnosis [41]. The mechanical ventilation involves an operation mode that incorporates 4 basic parameters: pressure or volume, fraction of inspired oxygen (FiO2), respiratory frequency, and expiratory pressure [38]. 

Based on the cycle between the inspiratory and expiratory phases of breathing, ventilators can are classified as follows: (i) volume cycled, where the ventilator cycles between inspiration and expiration once a preset tidal volume is achieved; (ii) pressure cycled, where the ventilator cycles between inspiration and expiration once a preset inspiratory pressure is reached; and (iii) time cycled, where the ventilator cycles between inspiration and expiration once a preset inspiratory and expiratory time has elapsed [41].

Mechanical ventilators have the following elementary units [42]: (i) Inlet regulation gas unit, composed of regulating valves to maintain gas pressure, oxygen, and compressed air within the working levels of the equipment. (ii) Blender, which is responsible for mixing the gases within the oxygen concentration reference range, which varies from 21 to 100%. (iii) Microcontroller (or Control unit), which configures the equipment in the desired functions, controls all other units, and manages the alarms and data of the device. The control valves are essential elements in the control unit, in addition to the respective flow and pressure sensors, which are also directly connected to it. (iv) Power source unit, which is responsible for supplying the system with energy from the electric network or from backup batteries. (v) Human interface unit, which includes a display, a touchscreen, knobs, and a keypad. This unit facilitates communication between the ventilator and the operator. (vi) External circuit (patient circuit), which is responsible for “connecting” the patient to the machine through the endotracheal tube and producing inspiration and expiration cycles via exhalation valves.

**Figure 2:**
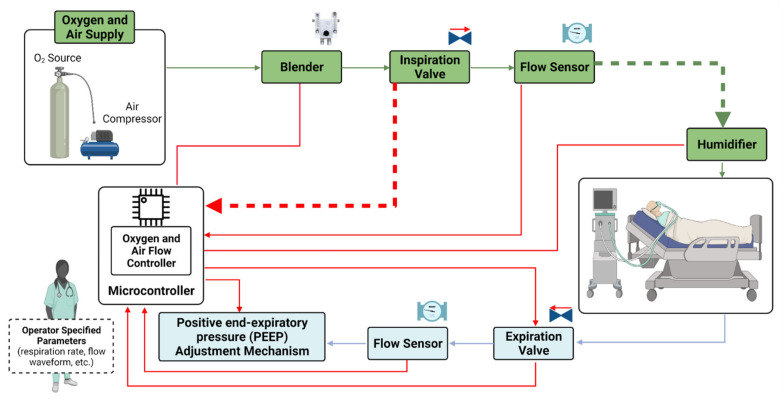
Block diagram of mechanical ventilator showing main parts and operation principle (Adapted from Ref. [43]).

A block diagram of a basic ventilator is shown in Figure 2. The air and oxygen sources are connected to valves and flow sensors. The microcontroller monitors the output of the flow sensors and controls the valves accordingly. In this way, the ventilator produces oxygen-enriched air with a specified oxygen concentration and conducts it to the tank. During inspiration, the ventilator opens the inspiration valve and closes the expiration valve. The inspiration valve is controlled in so that the patient receives breaths consistent with the desired predefined waveforms [43]. 

The reference unit received a total of 29 different ventilators models from 16 different manufacturers. These numbers show the complexity of the operation and of the standard procedures implemented in an emergency way to repair the ventilators.

### 2.2. Maintenance Collaborative Network

The voluntary network is composed of 39 maintenance sites distributed in 21 states of Brazil and is supported by the Ministry of Health, Ministry of Economy, Ministry of Defense, Brazilian Agency for Industrial Development (ABDI), and Brazilian Association of Clinical Engineering (ABEClin). The network was formed by 24 institutes of SENAI; 20 private companies (ArcelorMittal, BMW Group, Fiat Chrysler Automotive (FCA), Globo Studios, Ford Motor Company, General Motors, Honda, Hyundai Motor Brazil, Votorantim Institute, Jaguar Land Rover, Mercedes-Benz Brazil, Moto Honda, Renault, Scania, Toyota, Troller, Usiminas, Vale, Volkswagen Brazil, and Volvo Brazil); 3 universities and technology institutions (Institute of Technological Research (IPT), University of São Paulo (USP-POLI), and Federal University of Espirito Santo (UFES)); and 1 federal foundation (Oswaldo Cruz Foundation (Fiocruz)).

**Figure 3:**
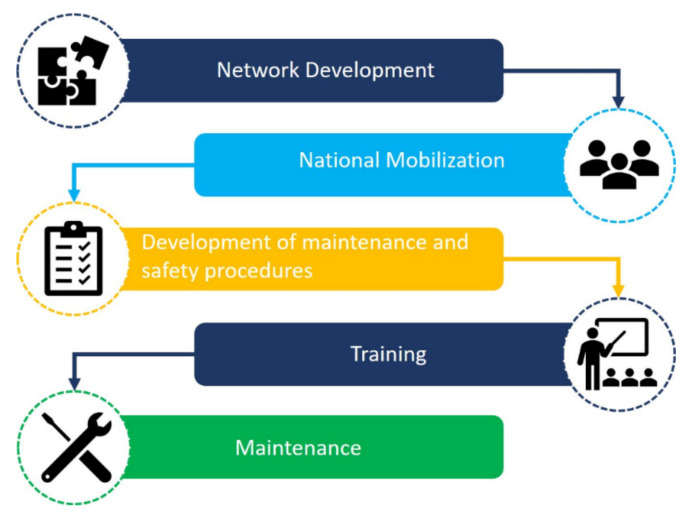
Flowchart of the main stages followed to structure the collaborative network for maintenance of ventilators.

Located in the state of Bahia, the Reference Unit was the first maintenance site to start operation and led the implementation of the network through the design of protocols, establishment of procedures, and provision of training for all teams around the country. The aim was to transfer knowledge and accelerate the structuring of the network. In order to disseminate the methods and procedures, a series of national and international events were carried out through online platforms. Figure 3 shows a flowchart with the main stages of structuring the collaborative I+M ventilator maintenance network.

### 2.3. Maintenance Process Lines

**Figure 4:**
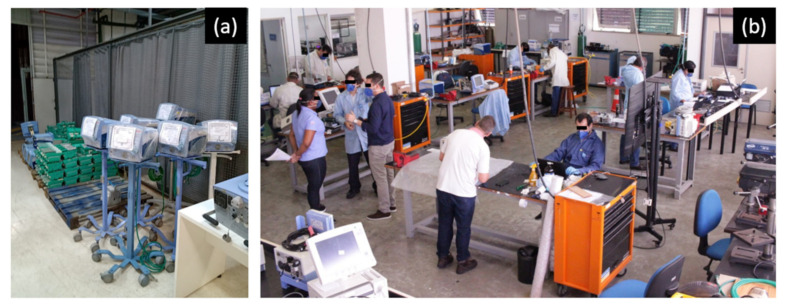
Main workspaces of maintenance line of ventilators implemented at Reference Unit: (**a**) receiving, cleaning, and disinfection area, and (**b**) general maintenance area.

The maintenance at the Reference Unit was performed mainly in two working spaces, as shown in Figure 4a,b. At first, the ventilators were received in an area that complied with the quarantine, cleaning, and disinfection rules, with around 100 m² of space, organized with pallets to store the equipment during quarantine (Figure 4a), benches for cleaning and disinfection, benches for working in process storage, and an internal cleaning and disinfection room equipped with air compression and filtration systems. The reception working space was ion the ground floor to facilitate the logistics and sufficiently ventilated to avoid manipulating healthcare equipment in a closed place.

The second workspace (Figure 4b) was structured to allow ventilator maintenance to be performed. The laboratories, about 100 m² in size, were equipped with maintenance tool kits (mechanics end electronics), pneumatic systems, rubberized benches with power points and compressed air, oxygen cylinders with regulator valves and hoses to distribute the gas for each bench, compressed air central filtration system, medical air compressor, gas flow analyzers for ventilator measurement and calibration, artificial lungs, a 3D printer, and a water sink. 

In addition to the equipment and infrastructure, it was also essential that the areas allowed for organization of the material and information (data) flow to ensure reliability and efficiency in the maintenance process. The guidelines also included flowcharts of the processes, an organization chart of the team, procedures for pickup and delivery operations, traceability tools for each ventilator during its stay at the maintenance site, external and internal logistics, purchasing process, and stock management and dashboards to facilitate communication with the team.

In all areas, rules, guidelines, and materials related to health, safety, and environment (HSE) were also in place. The use of protective equipment, such as masks, face shields, coats, and gloves, as well as daily HSE meetings and scheduled reverse transcription polymerase chain reaction (RT-PCR) tests were mandatory.

The administration and technical teams included employees of the maintenance site and volunteers, including specialists and technicians in medical and hospital equipment, clinical engineers, mechanical and electronic engineers, logistics engineers and analysts, project managers, safety technicians, purchase specialists, and communication and marketing analysts.

## 3. Results and Discussion

Although results from the whole network are referred to in this section, this work focused on specific results achieved by the Reference Unit maintenance site located in Bahia state. The challenges faced and results achieved were quite similar for most maintenance network sites.

### 3.1. Network Mobilization

The launch of the maintenance line was strategic planned by the Reference Unit based on the serious concern that most countries would experience a shortage of mechanical ventilators [22] and to prevent the collapse of health systems [38]. In general, the maintenance line implementation was aimed at repairing ventilators at public and private healthcare units that were out of operation and returning them ready for use, thereby contributing to increasing the number of ventilators available to patients with COVID-19. The challenge started including logistics (pickup to delivery), cleaning and disinfecting the ventilators when they arrived at the maintenance site, screening and identifying the operational issues, acquiring parts to replace broken ones, executing the repair, and calibrating the ventilators to meet operational parameters.

The first step was to establish a process and a team to map and get in contact with healthcare units that needed ventilator maintenance assistance, and plan the logistics to collect the equipment and generate a service order to control and ensure the correct return once the maintenance was completed. In the case of Bahia, according to the Bahia State Health Secretariat (SESAB), the service network involved in battling COVID-19 relied on 123 healthcare units. In terms of territorial extension, Bahia is the fifth largest state in Brazil, with 567,295 km^2^, and its road network is 119,639 km long. In total, the Reference Unit received 536 ventilators for repair between 3 March and 25 September 2020 and supported 58 cities and 7 mesoregions in Bahia state.

**Figure 5:**
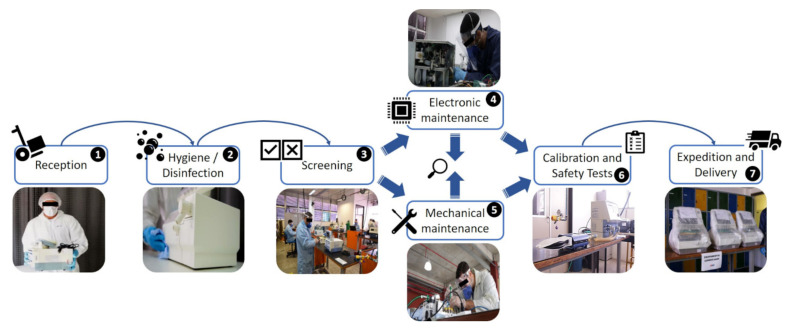
Flowchart showing all steps to complete ventilator repair, from reception to expedition and return to original healthcare unit. Maintenance line at Reference Unit.

Once the ventilator arrived at the maintenance site, a cleaning and disinfection protocol was initiated to allow the maintenance process to take place. Figure 5 presents the overall flowchart representing all the steps to complete the ventilator repair through to the expedition and return to the original healthcare unit.

The flowchart helped to standardize the protocols and procedures to be implemented around the country in order to ensure a safe and efficient maintenance process. All documents generated were distributed and used for training of all people involved. Here, a business continuity plan (BCP) was formulated to establish important rules and protocols following WHO guidelines [44], including the importance of washing hands and using masks, as well as a protocol to perform RT-PCR tests for 100% of the staff working at the site every two weeks. The steps and procedures developed for maintaining the ventilators and the control protocols for each stage are presented in Table 1. 

### 3.2. Training Sessions

Technical support was provided to every new maintenance site incorporated in the network. Training materials, operational ventilator manuals, and video classes were available to all members of the I+M. Different sorts of training were held; the initial training represented the beginning of the journey, so the dynamics necessary to carry out maintenance at the new sites could be understood, as well as the minimum infrastructure necessary to perform the project. There were 8 sessions that reached an audience of 100–160 people per session. The initiative sparked interest in other countries, thus it was adapted for Latin America, Africa, and WorldSkills member countries.

Another sort of training was live broadcasts from specialists and clinical engineers to present specific ventilators and answer technical questions from the members of the network. The “Conversation with a Specialist” training sessions had a total live audience of 187 participants and all the videos were made available on YouTube, with more than 5900 viewers. Table 2 presents the details of the sessions held and numbers of participants and viewers up to 15 March 2021.

The nationwide events were carried out using an online platform. The international trainings were broadcast via the Reference Unit’s official YouTube channel. All documents, procedures, and protocols generated were made available on a communication platform so that the technical teams at the maintenance sites could have access to implement the information in their processes during the I+M period. The open-source operation manuals are also available on the ANVISA website [48]. Details are described in Appendix A. It is important to note that all guidelines and information distributed followed the regulations and best practices of Brazilian clinical engineering (Table 1) and received the approval/validation of the clinical engineers who supported the process.

The training sessions proved to be a fundamental strategy to quickly empower the maintenance sites, contributing to increasing the number of ventilators successfully repaired. In total, 2516 ventilators were successfully repaired and returned to healthcare units, from 4047 ventilators received by the maintenance sites, which represents a success rate of 62.17%.

The videos and technical live sessions discussing critical cleaning and disinfection protocols and procedures proved to be essential in creating a safer and heathier environment for all participants. In addition, the strategy to centralize all technical information in a hub allowed the network to have updated documents and technical information, crucial for the success and feasibility of the initiative.

### 3.3. Network Mobilization

Besides all of the effort to rapidly launch the network, another challenge was to finance the new parts and components needed to replace damaged ones. Technological advances in the manufacture of mechanical ventilators with the incorporation of microprocessors and advanced features have led to significant improvements in this kind of equipment [49]. Along with these advances, the number of different mechanical ventilator modes makes the process of maintaining and managing such equipment more difficult, mainly in acquiring components and materials on the international market [50]. Thus, efforts to restore old and inoperative ventilators, as well as portable ones and those used in anesthesiology [51], became a key factor for the success of the planned strategy.

**Figure 6:**
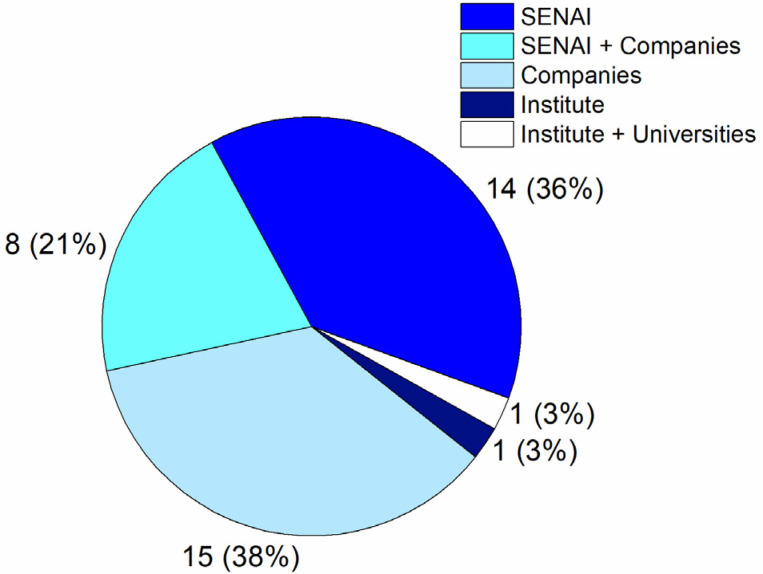
Distribution of institutions by maintenance unit. Among 39 maintenances units, SENAI institutes were involved in 59%.

As new members joined the I+M initiative, the project received more attention from additional companies/institutions for funding. As already mentioned, the last network report already counted 39 maintenance sites present in almost all states of Brazil, mobilizing in 53 institutions among SENAI units, companies, universities, foundations, and institutes. Figure 6 shows the distribution of institutions across the maintenance sites. It can be seen that institutes of SENAI were present at 23 maintenance sites, which corresponds to 58% of units involved in the I+M initiative. It must be highlighted that among all participating companies, 81.5% are considered large companies, and most of them are related to the automotive sector.

Additionally, another 18 companies and institutions, both private and government participants, supported the initiative with further funding. Their names have not been released for confidentiality reasons.

**Figure 7:**
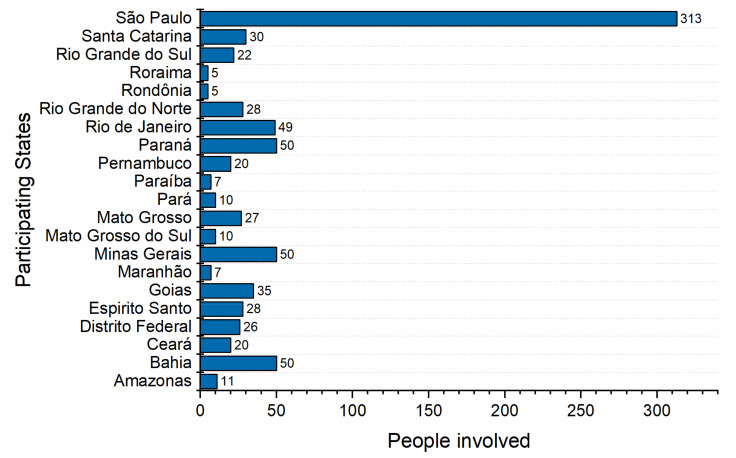
Distribution of people involved in I+M initiative per state of Brazil.

The I+M initiative mobilized more than 800 people directly involved in the maintenance activities. Among these were mechanical, electronic, and clinical engineers, as well as technicians and specialists. Figure 7 shows the distribution of people involved by federal state in Brazil. It can be noticed that the state of São Paulo mobilized the largest number of people (313) and the others had an average of about 25 people. Clearly, the number of professionals involved is directly related to the number of mechanical ventilators received, which will be discussed in the next section.

### 3.4. Maintenance Numbers

The I+M initiative presented its final report on 15 December 2020. The numbers of mechanical ventilators received and repaired are presented in Table 3. It can be seen that the success rate of the whole network was 62.17%, and it varied for each state. This variance can be attributed to factors such as the state of degradation of the ventilators upon arrival at the maintenance sites and the availability of spare parts on the market. It must be mentioned that the values do not represent the success rate by individual maintenance units; in some cases the sites received ventilators from other states.

**Figure 8:**
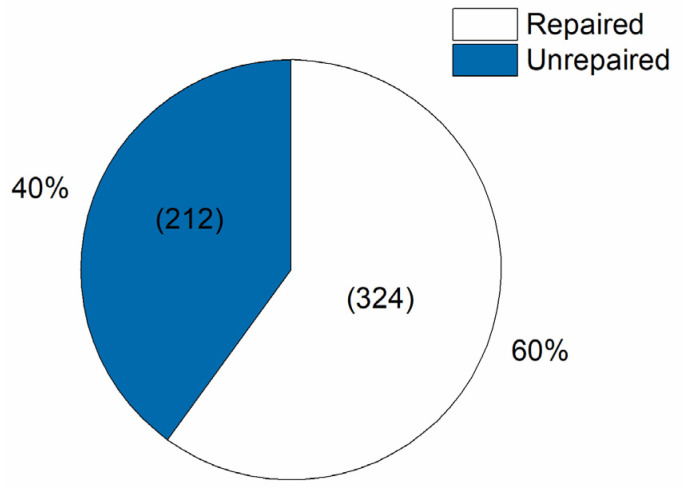
Number of ventilators repaired and not repaired at Reference Unit.

The Reference Unit maintenance site received a total of 536 ventilators from five states, 439 ventilators being sent only by the state of Bahia (279 recovered) (Table 3), 41 from Rio de Janeiro (5 recovered), 28 from Mato Grosso do Sul (25 recovered), 22 from Mato Grosso (9 recovered), and six from Rondônia (6 recovered). Of this total of 536 ventilators, 324 were repaired, for a success rate of 60%. The other 212 ventilators were classified as unserviceable, as they did not have the minimum amount of sufficient parts to perform the repair. Figure 8 presents the numbers and percentages of ventilators repaired and not repaired at the Reference Unit. The success metric for the Initiative was the number of ventilators repaired, which the initial target was 180 ventilators for the Reference Unit, considering that the project would last 3 months. Since the institutions gradually joined the network, it was not possible to establish a global target for the entire Initiative since the beginning.

It is important to emphasize that this was an unprecedented initiative with regard to the maintenance of ventilators and no similar initiative, whether public or private, was carried out. Thus, without the support of this maintenance network, it is believed that it would not be possible to achieve this number of repaired ventilators.

**Figure 9:**
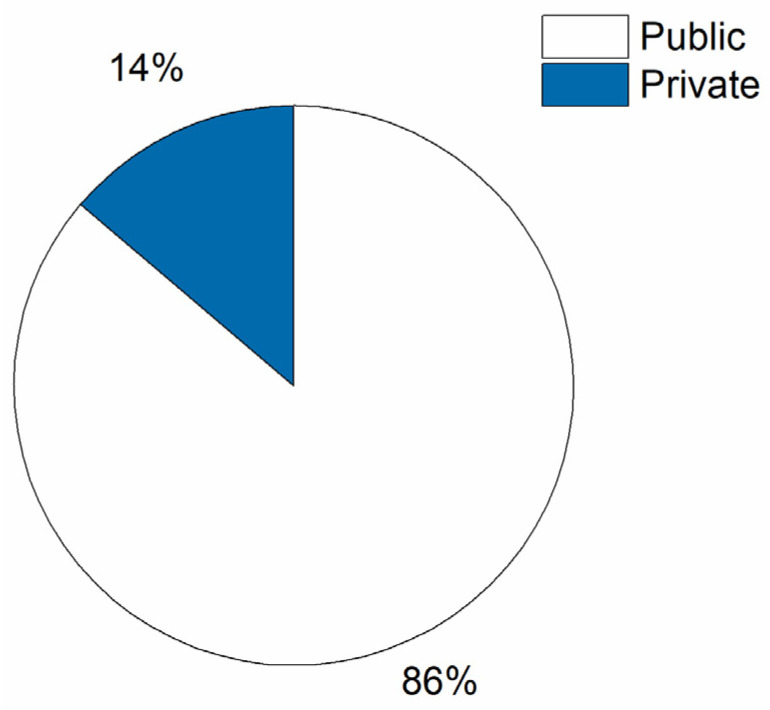
Public and private hospitals attended by I+M initiative in Bahia state.

As already mentioned, the initiative received mechanical ventilators from public and private healthcare units. In the case of the Reference Unit, the majority of ventilators came from public healthcare units, as shown in Figure 9. Another important aspect is related to the geographic locations of the hospitals and healthcare institutions. Most of them are located in the countryside and have very few ventilators available for the local community, which are often out of service. This supports the importance and relevance of the I+M initiative.

**Figure 10:**
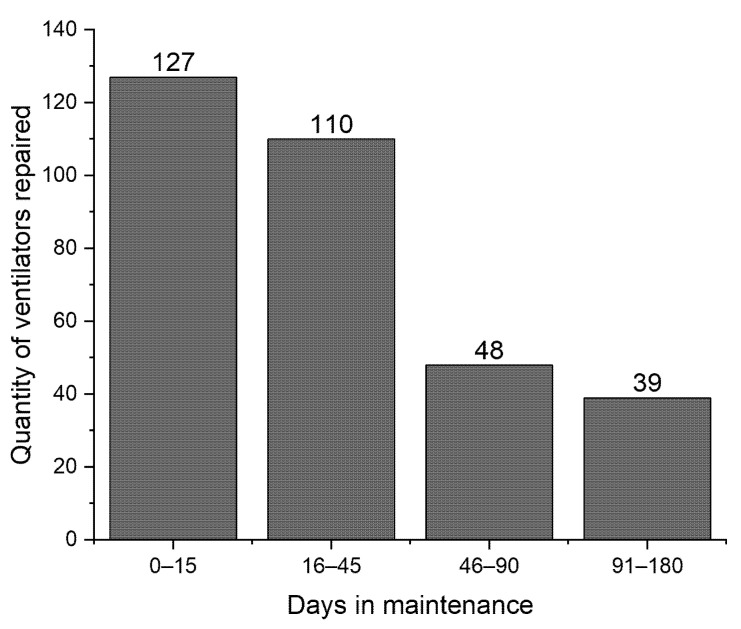
Number of ventilators repaired per time period at Reference Unit.

The lead time to repair the ventilators, considering pickup, cleaning, disinfection, screening, spare parts acquisition, maintenance, tests, calibration, and returning to the healthcare units, was one of the biggest challenges for the maintenance sites. As presented in Figure 10, during the I+M initiative period, the Reference Unit maintenance site repaired more than 73% of mechanical ventilators in less than 45 days. The repairs that exceeded this time were for ventilators that depended on parts supplied by manufacturers or representatives of manufacturers.

Although the Reference Unit produced a relevant number of repaired ventilators, as presented in Figure 10, long lead times (greater than 60 days in some cases) for supplying spare parts was experienced by the I+M network, especially for imported parts and systems. It was very common that consumables such as flow and oxygen sensors for specific models were unavailable on the market. The average time for receiving spare parts and consumables after starting the purchase process on the market was around two weeks. This became a critical factor, since it was necessary to place a large number of purchase orders to meet the constant demand for parts and accessories for ventilators received for maintenance.

**Figure 11:**
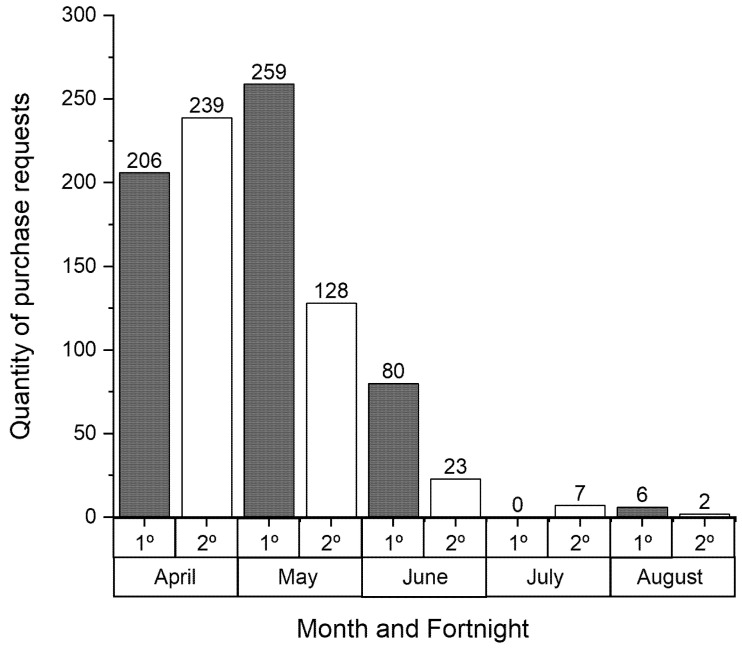
Number of part purchase orders per fortnight between April and August at the Reference Unit.

Figure 11 shows the number of purchase orders launched every two weeks by the Reference Unit. A total of 950 purchase orders were placed in the five month period of the initiative. The management of and continuous diligence in the purchase process were important factors for the success of the initiative.

In addition, due to contract rules, some manufacturers were only able to supply specific parts and components directly to the health units, which also affected the progress of the activities of the maintenance sites. For the 324 mechanical ventilators repaired by the Reference Unit, a total of 508 spare parts were used, resulting in an average of two items exchanged by equipment. The average expenditure per piece of equipment was USD 662.52.

According to a report by CGU, a Brazilian public financial office that regulates the use of public resources, published in May 2020, states and cities spent an average of USD 16,000.00 for every new mechanical ventilator [52]. Despite the lack of availability in the global market and considering that the I+M network repaired 2516 ventilators, and considering an average investment of approximately USD 1570.00 per ventilator including all expenses, such as: volunteer hours contributed, spare parts, fuel and transport, RT-PCR tests and disinfectants used, between other, which totals an initiative cost around USD 4 million, it would be reasonable to assume that the initiative saved around USD 36 million in public funds.

Since the beginning of the pandemic, there have been concerns by health authorities that the amount of mechanical ventilators available for the treatment of COVID-19 was less than the demand for these devices, as the virus spread rapidly [53]. The shortage of ventilators was observed in healthcare facilities in different parts of the world, including developed and developing countries such as the United States, Italy, Brazil, and countries on the African continent [23,54,55]. Within this context, different initiatives were adopted to mitigate the impact of the low availability of these devices in health systems, including granting emergency use authorization for new ventilators [38,56], establishing collaborative networks between countries for local production of the equipment [57,58,59], and scaling up the production of new low-cost and effective ventilators to keep up with the high demand [60,61,62]. To this end, some recognized ventilator manufacturers reallocated human, financial, and material resources to increase production of the devices, in addition to allowing free access to the ventilator designs so that other engineering companies could produce and market them [63,64]. The COVID-19 pandemic allowed for advances in the development of mechanical ventilators, considering the use of widely available materials and simple manufacturing techniques, which may be considered as progress, even to face new epidemics [65].

In Brazil, ANVISA is responsible for the registration of ventilators, which considers, among other steps: viability and regularization of the company; control and project development; the conformity certification and the product registration. This is a bureaucratic and time-consuming process in normal times. Due to the pandemic scenarios, ANVISA has been adopting measures to flexibilize the ventilators registration, such as the technical note 356/2020, which simplifies and streamlines the medical equipment regularization processes [66].

Another aspect that influenced the complexity of maintenance was the great diversity of manufacturers and models available in the Brazilian healthcare system. Despite their general similarities, each model has specific characteristics that, in many situations, demands a longer time to diagnose the defect and repair the equipment.

**Figure 12:**
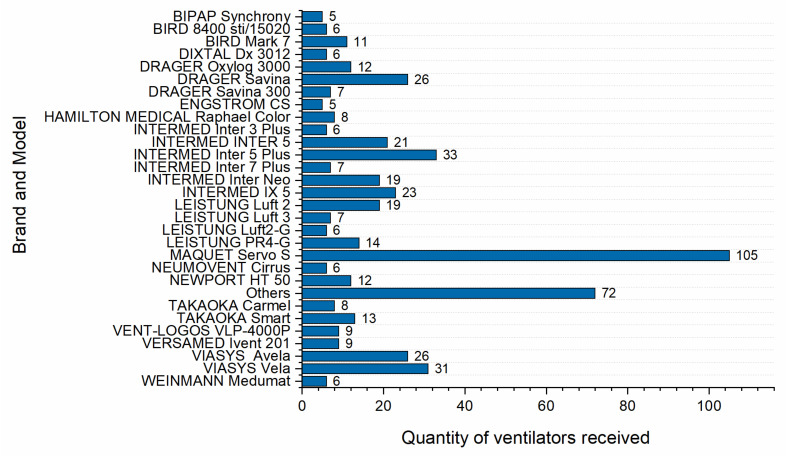
Number of ventilators received by brand and model at the Reference Unit.

Figure 12 shows the total number of ventilators received at the Reference Unit by brand and model. A total of 29 distinct models were received from 16 different manufacturers. Due to this, it was necessary to have more intersecting knowledge, beyond the difficulty of having access to information from the respective manufacturers, most of which is restricted to the general public.

During the period of the I+M initiative, the Reference Unit set up one workspace to receive, clean, and disinfect the mechanical ventilators and three maintenance laboratories, thus increasing its capacity to receive and recover more ventilators, so it also expanded the team dedicated to the project. The low availability of specialized technicians and engineers with knowledge of mechanical ventilation systems and equipment was an important bottleneck that the I+M initiative had to deal with.

Despite the restrictions brought about by the pandemic, the initiative counted a representative number of professionals who volunteered to participate. In the case of the Reference Unit, at its peak, it had 37 people on the technical team focused on equipment repair, in addition to the logistics, management, and purchase teams.

This productivity performance was also the result of applying tools from the lean philosophy, the principle of which is to do more with less waste, time, and resources [67]. Adopting practices such as continuous flow, 5S, Kanban, golden zone, inventory management, standardization of processes, cash management, performance dialogue, and Kaizen was essential to organizing the entire flow of materials, information, people, and finances during the project.

In total, the I+M initiative successfully repaired 2516 ventilators and returned them to healthcare units. Considering a theoretical number calculated at the maximum continuous capacity and assuming that, on average, a patient will require the continuous use of a mechanical ventilator for 15 days, in 6 months of use, the I+M initiative helped medical doctors and hospitals to support 30,192 human lives with mechanical ventilation.

It is important to emphasize that, in an unprecedented way, other initiatives were observed around the world, such as the development of new ventilators that were easy to manufacture [68,69]; the maintenance and production of medical hardware to help treat COVID-19 patients using 3-D printers [70] or even just building adapters to share ventilators and thus increase capacity [71].

## 4. Conclusions

In this work, an overview of the rapid deployment of a network for maintaining disused mechanical ventilators in public and private healthcare units is presented. The data show that the maintenance I+M at the Reference Unit had a large positive impact, covering more than five Brazilian states and directly affecting the treatment of a great number of patients. Many technological and logistical challenges were overcome. The implementation of the first line of ventilator maintenance in three days, meeting the protocols and norms, in partnership with clinical engineering professionals was a great challenge. Logistical structuring, from collecting ventilators to returning them to health units and developing procedures for cleaning and disinfecting equipment, was another great issue, in addition to the training of specialized labor for this type of action.

It is of fundamental importance to establish complementary criteria for the routine maintenance and management of spare parts for ventilators in public and private health networks, to ensure that ventilators remain usable for a long period of time. These aspects have a direct impact on the availability and reliability of equipment, and consequently on the ability to assist patients in treatment. In summary, the report provides the following information:

- The first maintenance unit was launched in three days with all required protocols and safety
- The engagement of SENAI institutes in the initiative allowed quick setup of new units once the institutes were present in all states in Brazil. Additionally, the interest of volunteers and industrial companies in supporting the initiative allowed knowledge transfer and financial support.
- The 2516 ventilators the initiative returned to healthcare units supported 30,192 human lives in the worst situation of the COVID-19 pandemic, considering a theoretical number calculated at the maximum continuous capacity.
- There was support in improving the infrastructure of the state hospital network and training of specialized labor for this type of action.
- The biggest challenges were the logistics, specialized manpower, and availability of spare parts to perform the maintenance.

## Figures and Tables

**Table 1:** Steps and procedures developed by Reference Unit.

Procedures	Description	Documents
Reception	Receipt and registration of ventilators. Protocols and procedures were defined in terms of necessary personal protective equipment (PPE) as well as the need for a photographic registry and registration in the warehouse management system (WMS). This step was important to ensure that ventilators and accessories were properly returned to healthcare units and to establish first in, first out (FIFO) process in maintenance line.	Receiving protocol
Hygiene/Disinfection	In this step, specific rules for maintenance lines were documented, validated, and distributed to personnel involved. Some rules defined that: a. External cleaning and disinfection of equipment should be carried out with 70% isopropyl alcohol. b. Breathing circuits (ventilator endotracheal tubes) should be disassembled and sanitized with 70% ethyl alcohol and sent in sealed plastic bags to a biotechnology laboratory for autoclave decontamination. c. After external cleaning, ventilator should undergo 48 h quarantine to ensure decontamination of internal parts. d. Equipment should be opened in a room with an exhaust fan fitted with a bag filter (F9) and an absolute filter (F772 or F782).	Basic cleaning protocol
Screening	At this step the objective was to identify equipment issues by evaluating and comparing with operational end service manuals. Many ventilators needed consumables such as batteries and oxygen cells replaced, but in order to evaluate their parameters, tests should include assembly of endotracheal tube circuits and initial calibration test should be run. Performance at initial trial generally demonstrated, through alarms, actions to be taken. Qualified technical teams, including clinical engineers and biomedical equipment technicians, and infrastructure, including oxygen and compressed air cylinders, artificial lungs, and gas flow ventilator analyzers, were crucial at this point forward.	Operation and service manuals
Electronic maintenance	Once the need for electronic repair is identified, the ventilator should be examined at this stage to repair defects, such as damaged electric key and sensors. Importantly, all ventilators were in use by healthcare units; consequently, their use was approved by the National Health Surveillance Agency (ANVISA). Thus maintenance did not change any aspect related to ventilator design, such as printed circuit board design or component specifications.	Basic requirements for ventilator maintenance
Mechanical maintenance	Most malfunctions were related to electronics and consumables, but in some cases, ventilator models were primarily based on a mechanical operating mechanism. It was usual to find punctured and dry hoses in such ventilators. After electronic and/or mechanical repair, calibration pre-trial was performed to ensure maintenance was done well.	Basic requirements for ventilator maintenance
Calibration and safety tests	A fundamental step in the maintenance process was final calibration and electrical safety tests. Calibration followed guidelines and procedures based on ABNT NBR ISO/IEC 17025:2017 standard [45]. Electrical safety followed ABNT NBR IEC 60601-1 [46] and ABNT NBR IEC 62353 [47] standards. This step ensured that every ventilator, once approved, met expected performance and posed no risk when used by hospitals. It was very important that healthcare units used the equipment according to operation manuals.	Operation and service manuals
Expedition and delivery	Final step, including cleaning to ensure that oldest tags were removed and review of all accessories according to ventilator’s entrance registration and packing. Upon completion, the logistics team was triggered to return ventilators to healthcare units as soon as possible.	Devolution protocol

**Table 2:** Main training and knowledge transfer events.

Type	Goal	Target Audience	Coverage	Workload (Hours)	Quantity	Number of Participants/ Viewers
Initial training	Demonstrate knowledge, standards, safety procedures, and basic infrastructure to perform ventilator maintenance	SENAI institutes and partner companies	National	4	8	803
International live		Companies in Latin America, Africa, and USA	International	2	3	617
Conversation with a specialist	Transfer specialized knowledge of ventilator maintenance focusing on most common brands/models	Maintenance sites (I+M)	National	1	3	187
Clinical engineering live	Discuss strategy and future developments in clinical engineering in Brazil	All partners and interested public	International	2	1	605
Question sections	Specific sections to answer questions regarding maintenance process and equipment	SENAI institutes and partner companies	National	1	3	08

Presentations available on: https://www.youtube.com/watch?v=0DHysjNTa2k (accessed on 15 March 2021).

**Table 3:** Distribution per state in Brazil of ventilators received and repaired (success rate).

State of Brazil	Number of Ventilators Sent	% of Ventilators Sent	Number of Ventilators Repaired	% of Ventilators Repaired
Acre	8	0.20	6	75.00
Alagoas	10	0.25	3	30.00
Amapá	17	0.42	13	76.47
Amazonas	40	0.99	26	65.00
Bahia	439	10.85	279	63.55
Ceará	127	3.14	110	86.61
Distrito Federal	104	2.57	71	68.27
Espírito Santo	88	2.17	65	73.86
Goiás	198	4.89	118	59.60
Maranhão	25	0.62	16	64.00
Mato Grosso	153	3.78	83	54.25
Mato Grosso do Sul	137	3.39	103	75.18
Minas Gerais	559	13.81	281	50.27
Pará	128	3.16	55	42.97
Paraíba	82	2.03	39	47.56
Paraná	146	3.61	52	35.62
Pernambuco	89	2.20	32	35.96
Rio de Janeiro	296	7.31	124	41.89
Rio Grande do Norte	44	1.09	40	90.91
Rio Grande do Sul	168	4.15	120	71.43
Rondônia	9	0.22	6	66.67
Roraima	28	0.69	20	71.43
Santa Catarina	79	1.95	54	68.35
São Paulo	1018	25.15	753	73.97
Tocantins	55	1.36	47	85.45
Total	4047	100	2516	62.17

## Data Availability

Data supporting reported results can be found on https://doi.org/10.6084/m9.figshare.14737848 (accessed on 5 June 2021).

## Appendix A

**Table A1:** Procedures, documents, and manuals.

Procedures	Documents	Link
Reception	Receiving protocol	https://doi.org/10.6084/m9.figshare.14737848 (accessed on 5 June 2021)
Hygiene/Disinfection	Basic cleaning protocol	https://doi.org/10.6084/m9.figshare.14737848
Screening	Operation and service manuals	Operations Manuals: https://consultas.anvisa.gov.br/#/saude/ (accessed on 5 June 2021) Service Manuals: Available for the Open Project Medtronic PB560. Link: https://www.medtronic.com/us-en/e/open-files.html (accessed on 5 June 2021) For other ventilators, please, consult the manufacturer.
Electronic maintenance	Basic requirements for ventilator maintenance	https://doi.org/10.6084/m9.figshare.14737848
Mechanical maintenance	Basic requirements for ventilator maintenance	https://doi.org/10.6084/m9.figshare.14737848
Calibration and safety tests	Operation and service manuals	Operations Manuals: https://consultas.anvisa.gov.br/#/saude/ Service Manuals: Available for the Open Project Medtronic PB560. Link: https://www.medtronic.com/us-en/e/open-files.html (accessed on 5 June 2021) For other ventilators, please, consult the manufacturer.
Expedition and delivery	Devolution protocol	https://doi.org/10.6084/m9.figshare.14737848

**Table A2:** Training and knowledge transfer events.

Type	Link
International live	https://www.youtube.com/watch?v=ELX_tzKlalQ (accessed on15 March 2021) https://www.youtube.com/watch?v=0DHysjNTa2k (accessed on 15 March 2021) https://www.youtube.com/watch?v=Uy6DYu2pdvM (accessed on 15 March 2021)
Clinical engineering live	https://www.youtube.com/watch?v=7pOarJOt7vY (accessed on 15 March 2021) https://www.youtube.com/watch?v=QCd-NkFFKEA (accessed on 15 March 2021)
